# *Staphylococcus aureus*-dependent septic arthritis in murine knee joints: local immune response and beneficial effects of vaccination

**DOI:** 10.1038/srep38043

**Published:** 2016-11-30

**Authors:** Alessia Corrado, Paolo Donato, Silvia Maccari, Raffaella Cecchi, Tiziana Spadafina, Letizia Arcidiacono, Simona Tavarini, Chiara Sammicheli, Donatello Laera, Andrea Guido Oreste Manetti, Paolo Ruggiero, Bruno Galletti, Sandra Nuti, Ennio De Gregorio, Sylvie Bertholet, Anja Seubert, Fabio Bagnoli, Giuliano Bensi, Emiliano Chiarot

**Affiliations:** 1 GSK Vaccines, Via Fiorentina 1, Siena, 53100, Italy

## Abstract

*Staphylococcus aureus* is the major cause of human septic arthritis and osteomyelitis, which deserve special attention due to their rapid evolution and resistance to treatment. The progression of the disease depends on both bacterial presence *in situ* and uncontrolled disruptive immune response, which is responsible for chronic disease. Articular and bone infections are often the result of blood bacteremia, with the knees and hips being the most frequently infected joints showing the worst clinical outcome. We report the development of a hematogenous model of septic arthritis in murine knees, which progresses from an acute to a chronic phase, similarly to what occurs in humans. Characterization of the local and systemic inflammatory and immune responses following bacterial infection brought to light specific signatures of disease. Immunization of mice with the vaccine formulation we have recently described (4C-Staph), induced a strong antibody response and specific CD4+ effector memory T cells, and resulted in reduced bacterial load in the knee joints, a milder general inflammatory state and protection against bacterial-mediated cellular toxicity. Possible correlates of protection are finally proposed, which might contribute to the development of an effective vaccine for human use.

*Staphylococcus aureus* is a human pathogen responsible for a variety of diseases ranging from minor/mild skin infections to life threatening diseases^1^. It is a major cause of bacteremia, which frequently leads to severe complications like endocarditis, toxic shock syndrome, septic arthritis (SA) and osteomyelitis (OM)^2^. Among others, joint-related diseases deserve special attention because of their rapid evolution and serious clinical outcomes, such as intense pain and impairment due to bone erosion requiring urgent intervention^3^,^4^,^5^. Joint infections are frequently localized in the knees and hips, and monoarticular disease is more frequent and less severe than polyarticular infection^6^,^7^. The mortality rate associated with these infections is around 5–20% in the adult population^3^, but can reach 50% depending on delayed diagnosis, immunodeficiency, older age^3^,^4^ and pre-existing underlying conditions, such as rheumatoid arthritis and diabetes^3^,^8^,^9^. Joint and bone disruption is caused by the activity of bacteria in the joints, as well as by uncontrolled activation of the host immune system sustaining a local destructive inflammatory state, which can eventually result in chronic disease^10^,^11^,^12^,^13^. OM is often not treatable with antibiotics, a problem that is presently more evident with the increasing emergence of antibiotic-resistant *S. aureus* strains^14^,^15^,^16^,^17^,^18^,^19^. On the other hand, early application of antibiotic treatment can be efficacious for SA, which however often requires surgical intervention^3^,^4^,^7^,^20^.

Given these premises, the development of an efficacious vaccine able to prevent *S. aureus-*mediated SA and OM would be highly desirable. We have recently demonstrated that an adjuvanted protein combination vaccine, 4C-Staph, conferred significant protection against different *S. aureus* strains in multiple murine infection models, and that protection was dependent on both humoral and cellular immune responses^21^,^22^,^23^,^24^.

In this study, we report the long-term characterization of a hematogenous model of *S. aureus*-dependent SA and OM in mice. Animals were followed for as long as 3 months after the infection, showing chronic disease. During this period, the presence of bacteria was demonstrated in the knee joints, as well as in blood and kidneys, which were used as markers of systemic infection. A comprehensive analysis of cytokines and of different immune cell populations both in the sera and in the knee joints of infected mice provided the global picture of the elicited immune and inflammatory responses. Importantly, immunization of mice with alum-adjuvanted 4C-Staph vaccine reduced bacterial burden in the joints. In this context, we also demonstrated that 4C-Staph-specific antibodies played a role in protection and that CD4+ effector memory T cells expressing mainly Th0/Th2-associated cytokines were recruited at the infected joints.

## Results

### *S. aureus* systemic infection in mice results in fast knee joint infiltration which evolves to chronic disease

We have recently described a multi-protein vaccine formulation, 4C-Staph, which protects mice from *S. aureus* infection in different models^21^,^22^,^23^. Since arthrosynovitis and OM are among the most severe *S. aureus*-dependent diseases in humans^3^,^4^,^5^, we wanted to test the efficacy of the vaccine formulation in a mouse model of knee joint infection and evaluate local and systemic host immune responses.

**Figure 1:**
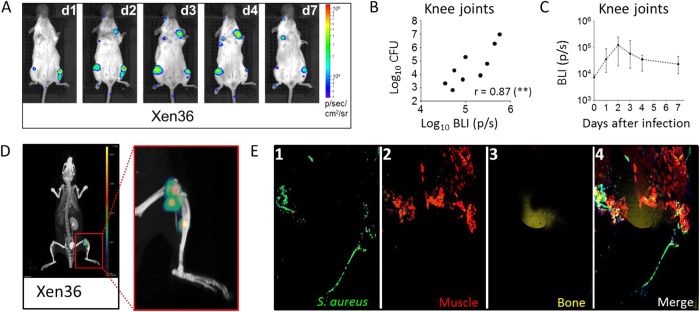
Intravenous injection of *S. aureus* in mice results in a rapid infection of bones and joints. (**A**) 2D Ventral pictures of a representative CD1 mouse intravenously infected with *S. aureus* Xen36 bioluminescent strain from day 1 (d1) to day 7 (d7) p.i. (**B**) Correlation analysis between CFU counts and BLI intensity at day 7 p.i. Spearman’s r value and significance are reported. (**C**) Quantification of the bioluminescent (BLI) signals belonging to the knee joint areas of infected animals from day 0 (naïve mice, before infection) to day 7. Median values of 5 animals at each time point are reported together with interquartile ranges. (**D**) 3D analysis of BLI signals from animals infected with Xen36 strain. A ventral picture of 1 animal out of 5 at day 2 p.i. is shown. In the panel on the right, an enlargement of the left hind paw is depicted. (**E**) Confocal microscopy image of a whole knee from one mouse infected with *S. aureus* Newman strain 7 days after injection. One out of 5 mice is shown.

*S. aureus* has been shown to have a particular tropism for bones and joints after intravenous injection in animals^25^,^26^. To initially confirm this, we used a bioluminescent strain to follow infection progression *in vivo* over time. Mice were intravenously infected with Xen36^27^ and bacterial spreading was followed for 7 days using an IVIS Spectrum-CT^®^ imaging system (Fig. 1A). Bioluminescent signals were detected in the knee joints as soon as 24 hours after infection (d1), persisting throughout and until the end of the 7-day observation period (d7). Interestingly, a good correlation was observed between bioluminescent signals and the number of colony forming units (CFUs) isolated from the joints (Fig. 1B). Since bioluminescence peaked at day 2 (Fig. 1C), computed tomography (CT) analysis was performed at this time point to better localize the infection site(s). The representative image in Fig. 1D shows that *S. aureus* infiltrated both the knee joint area and the bone. When other *S. aureus* bioluminescent strains from different capsular serotypes were used to infect mice, consistent signals were observed in the same areas (Fig. S1), suggesting that infection targets were independent from the strain used.

Since the bioluminescent strains required high infective doses (Xen36) or were not sufficiently bioluminescent (Xen29 and Xen8.1), we decided to use the non-bioluminescent Newman strain to better characterize the infection model, the host response to infection, and the protective potential of the 4C-Staph vaccine formulation. This strain was originally isolated from a case of secondary infected tubercular osteomyelitis, and has been previously used in the study of septic arthritis and osteomyelitis^28^,^29^. Confocal microscopy analysis confirmed the ability of Newman to localize at the joints following systemic infection of mice (Fig. 1E).

**Figure 2:**
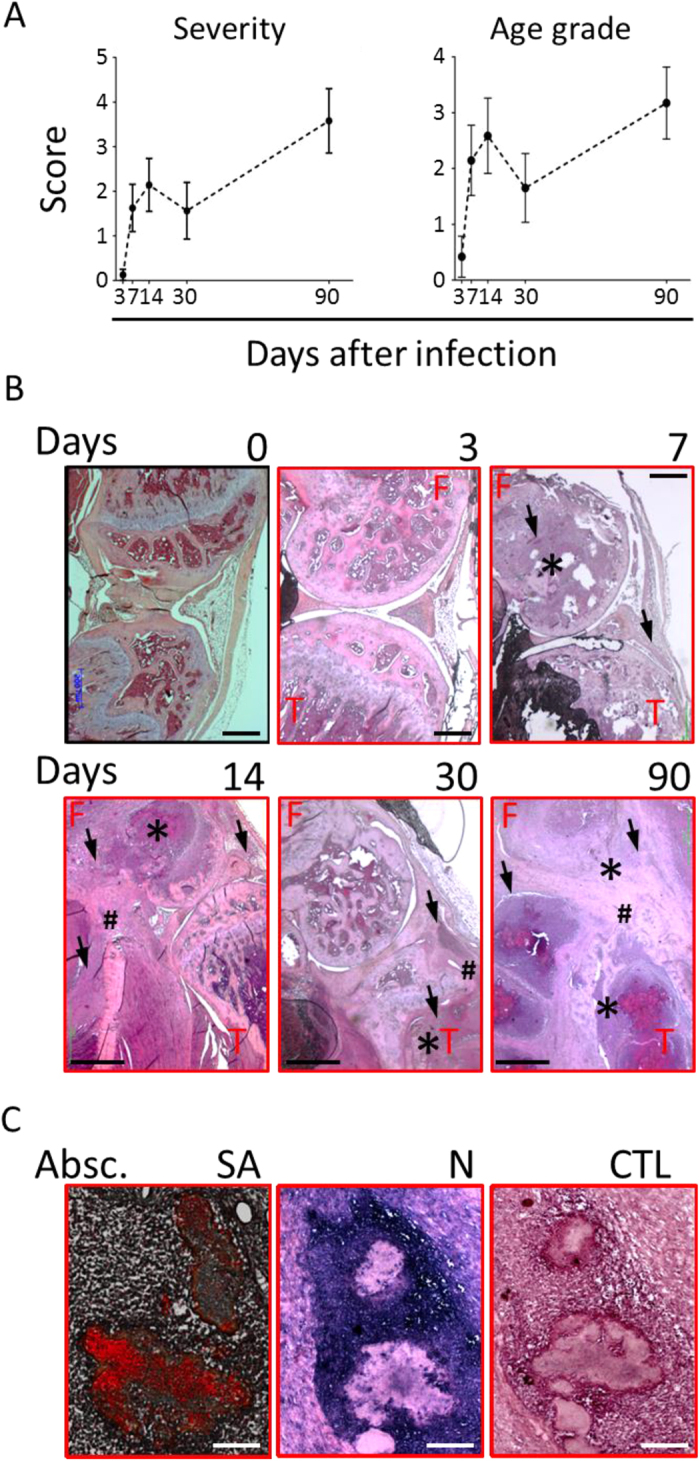
Mice intravenously infected with *S. aureus* develop arthrosynovitis and osteomyelitis, which evolve from an acute to a chronic state. (**A**) Histopathological analysis of the knees of infected mice. The knees from 4 to 6 animals/time point from 3 independent experiments were scored for severity of the lesions (Severity) and for progression of the infection (stage) both as to bones and joints analysis and mean +/− SEM were shown. (**B**) Representative hematoxilin-eosin-stained slide for 1 animal out of 4 to 6 at each time point. Time 0 is representative of naïve mice. Legend: F = femur, T = tibia, * = bone destruction/abscesses, # = fibroplasia/collagenous tissue, arrows = details of inflammatory cell accumulations. Scale bar: days 0, 3, 7, 200 μm; days 14, 30, 90, 1 mm; (**C**) Immunohistochemical staining of an abscess (Absc.) from an infected bone. *S. aureus* (SA, red specific staining, blue counterstaining) and neutrophils (N, blue specific staining, red counterstaining) were stained and a negative control (CTL) sample is shown in the third panel. Scale bar: 100 μm.

To evaluate disease progression and determine whether the infection could result in long-term persistence of bacteria in the joints as previously reported^25^,^26^, distinct groups of CD1 mice were intravenously infected with Newman (about 2E + 06 CFU/mouse) and were sacrificed at different time points, the latest being 90 days post-infection (p.i.). Histological preparations of infected knees at days 3, 7, 14, 30 and 90 p.i. (time 0 corresponded to non-infected mice) were analyzed and scored for both severity and stage of the lesions, based on presence and quality of inflammatory exudates and abscesses, bone destruction/necrosis, fibroplasia and accumulation of collagenous tissue. The results of these analyses are summarized in the graphs in Fig. 2A and detailed in Supplementary Table S1. After an initial, mild to moderate, acute phase of inflammation, which peaked at day 14 p.i., mice seemed to control the progression of the disease. But at day 90 p.i., both arthritis and OM worsened, showing a consistent presence of collagenous tissue, often surrounding abscesses and areas of bone destruction, which overall suggested a chronic disease. When inflammation was observed, bones (mainly femurs) and surrounding muscles were affected, while the involvement of the articular cavity declined with time. In Fig. 2B and C, representative slices are reported, showing the histological analysis of joints and abscesses.

**Figure 3:**
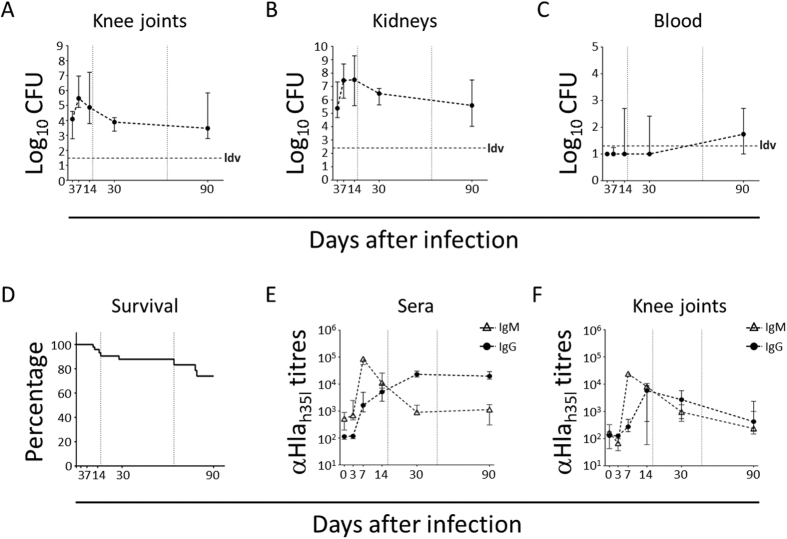
Infected animals are only temporarily able to control *S. aureus* dependent disease. (**A–C**) Log_10_ CFU counts recovered from the knee joints (CFU/organ, **A**), kidneys (**B**, CFU/organ) and blood (CFU/ml, **C**) of mice intravenously infected with *S. aureus* Newman strain from day 3 to day 90 p.i. Medians and interquartile ranges of 5 to15 animals/time point from 2/3 independent experiments are shown. ldv = lower detectable value. (**D**) Survival curve of mice intravenously infected with Newman strain. (**E,F**) IgM and IgG titration against *S. aureus* alpha-hemolysin_H35L_ (Hla_H35L_) in sera (**E**) and knee joint washes (**F**) of infected mice from day 0 (naïve animals) to day 90. Graphs report medians and interquartile ranges of Mean Fluorescence Intensity (MFI) signals of 6 to 8 mice at each time point and 2 independent experiments. In all the graphs reported above, the vertical dotted grey lines indicate the edges of the 3 proposed stages of infection.

CFU counts in joint washes were also evaluated, with the bacterial burden in kidneys and blood being used as a marker of systemic disease progression. As shown in Fig. 3A and B, a peak of bacteria was observed both in joint washes and the kidneys between days 7 and 14 p.i., before reaching a lower plateau at later time points, maintained until the end of the 90-day observation period. In contrast, bacteria were found in blood only at the 90-day time point (4 animals out of 8 observed, Fig. 3C). Alongside the local and systemic bacterial burden measurement, the general condition of mice was evaluated throughout the experiment to humanely sacrifice those showing pre-established clinical endpoints. Interestingly, mice survival was over 70% at the end of the 90-day observation period, during which two peaks of mortality were observed, the first around days 10–16, the second about 2–3 months after infection, with a stationary phase in between (Fig. 3D).

To obtain initial data reflecting host response to the infection, the levels of IgM and IgG specific for α-hemolysis (Hla) were evaluated in sera and knee joint washes of infected mice (Fig. 3E and F, respectively). As expected, IgM peaked very rapidly in sera (at day 7 p.i.) before decreasing over time, while IgG increased more slowly (peak at day 30 p.i.), reaching a plateau that was maintained during the following 60 days of observation. In the knee joints, both IgM and IgG peaked between the first and the second week after the infection and then progressively decreased.

### *S. aureus* hematogenous infection elicits local inflammatory and immune cell recruitment in the knee joints

Since kinetics of α-Hla titers differed in sera and the knee joints, we wanted to further dissect the host reaction to the pathogen evaluating the inflammatory cytokines and cellular immune responses.

**Figure 4:**
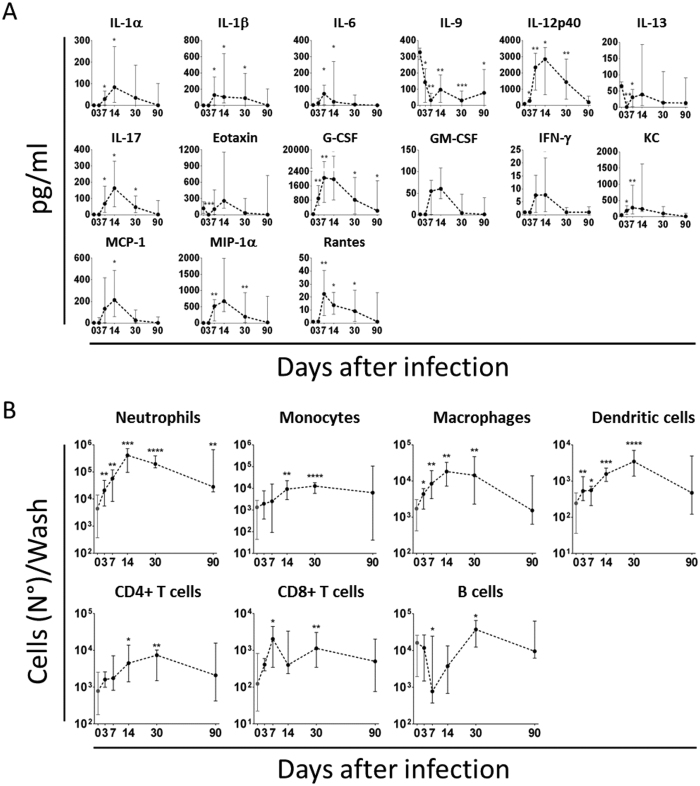
Rapid proinflammatory response and cellular recruitment follow *S. aureus* infiltration in the knee joints. (**A**) Cytokine profiles measured in knee joint washes of infected animals (pg/ml). Only cytokines which significantly changed with respect to the negative control (not infected animals, time 0) at least at one time point are reported here. (**B**) Phenotypic characterization of cells recruited into the knee joints of not infected (time 0) and infected mice after bacterial injection. The total number of each cell sub-population/wash is reported here. In both cases, samples were collected from 6 to 14 mice/time point from 3 independent experiments and data are reported as median values and interquartile ranges. Statistical analysis was performed using a two-tailed Mann-Whitney U-test comparing single time points against time 0.

Cytokines in knee joints, when measurable, increased, with few exceptions, between the first and second week p.i. (Fig. 4A), and then decreased to basal levels, somehow resembling the trend observed for the CFU counts (Fig. 3A). As a consequence, most cell types of the myeloid and lymphoid lineages increased in joint washes up to 30 days p.i. (Fig. 4B). The only exception was represented by B cells which showed a clear and sharp decrease in the first week after the infection, returning to basal levels within the first month of observation.

**Figure 5:**
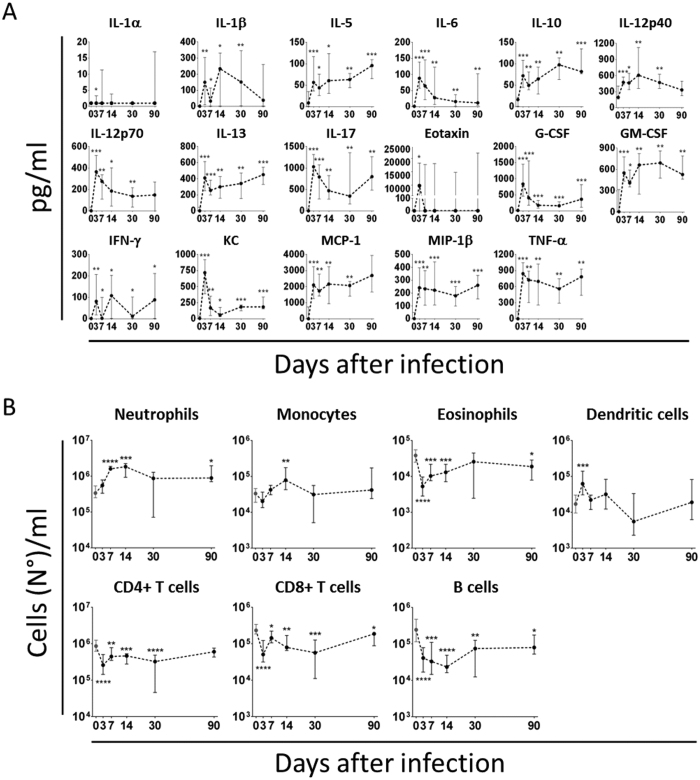
*S. aureus* systemic infection modulates host immune response in the blood. (**A**) Cytokine profiles measured in the sera of infected animals (pg/ml). Only cytokines which significantly changed with respect to time 0 at least at one time point are shown. (**B**) Phenotypic characterization of immune cells circulating in the blood of not infected (time 0) and infected mice throughout the observation period. Data were expressed as cell number/ml. In both cases, samples were collected from 6 to 14 mice/time point from 3 independent experiments and data were shown as median values and interquartile ranges. Statistical analysis was performed using a two-tailed Mann-Whitney U-test comparing single time points against controls.

The humoral and cellular situation in the blood was quite different and more complex. Several classical pro-inflammatory cytokines peaked very early after the infection (Fig. 5A), being however detected at lower levels throughout the entire observation period (e. g. IL-1β, IL-6, IL-12p40, IL-12p70, G-CSF and KC). Others remained at elevated levels up to 90 days p.i. without evident changes during disease progression (e.g. IL-5, IL-10, IL-13, GM-CSF, MCP-1, MIP-1β, TNF-α). Eotaxin was secreted only in the first stage of bacterial spreading, while IL-1α was hardly detected. Interestingly, IL-17 increased immediately after the infection, decreased during the following 30 days, and finally increased again in a later observation phase. Moreover, while the number of neutrophils and monocytes increased within 14 days p.i. (Fig. 5B), eosinophils and all the cells of the lymphoid lineage decreased shortly after the infection and generally remained at a constant, low level thereafter throughout the 3-month observation period. Dendritic cells showed a particular trend, increasing only at the earliest time point.

Overall, the data described so far demonstrated that a systemic *S. aureus* infection resulted in accumulation of bacteria in the knee joints, which in turn triggered a local inflammatory condition and the induction of a specific, chronically altered humoral and cellular response.

### The four-component vaccine 4C-Staph reduces *S. aureus* infection at the joints

**Figure 6:**
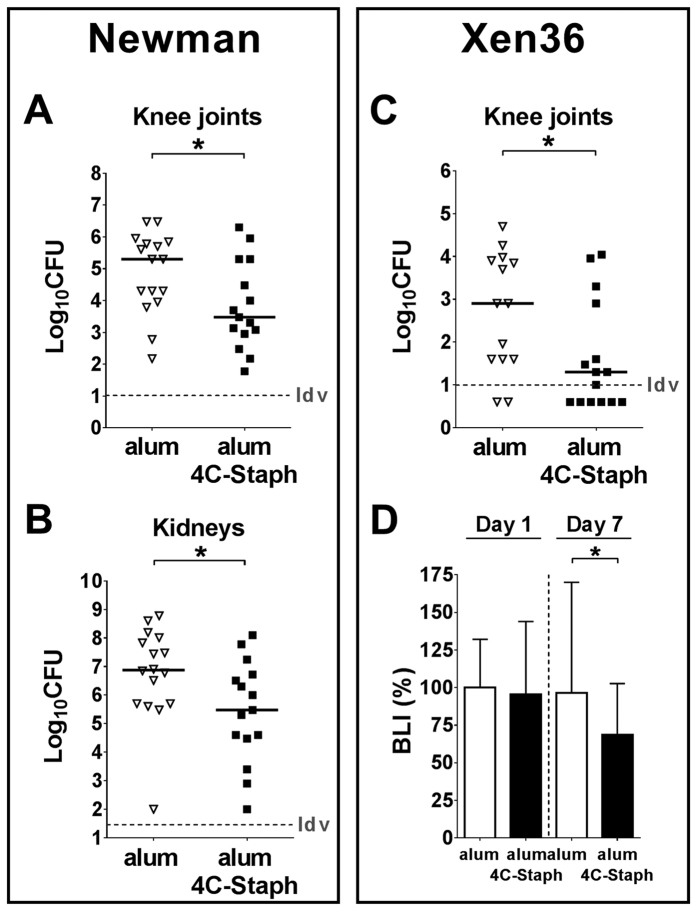
Immunization with the alum/4C-Staph protein combination results in reduction of bacterial burden in the knee joints of infected mice. (**A–C**) Log_10_ CFU counts of knee joint washes (**A–C**) and homogenized kidneys (**B**) of mice infected with Newman (**A-B**) or Xen36 strain (**C**). Mice were either immunized with alum/4C-Staph or alum alone (**A–C**). Single dots represent CFU counts of single animals from 2 independent experiments, the black horizontal line indicates median values while the dotted gray line is representative for the lower detectable value of the assay (ldv). (**D**) Normalized bioluminescent intensity (BLI) from knees of CD1 mice immunized with alum/4C-Staph or alum alone at days 1 and 7 p.i. with Xen36 strain. Columns and error bars represent medians and upper interquartile ranges, respectively. In both cases, the two-tailed Mann-Whitney U-test was always used to assess significance.

Once the *S. aureus* model of arthritis and OM was set up and characterized, we wanted to assess whether the 4C-Staph vaccine formulation, which we have recently described^21^, would be able to reduce bacterial load in the knee joints in the acute form of SA. When mice were immunized twice with 4C-Staph adjuvanted with aluminum hydroxide, and challenged intravenously with *S. aureus* Newman strain, a significant reduction in CFUs was observed 7 days after the infection, as compared with mock-immunized animals, both in knee joint washes (Fig. 6A) and the kidneys (Fig. 6B). We wondered whether the lower bacterial burden observed at day 7 in the joints of vaccinated mice was due to bacterial neutralization in blood within the first hours after infection, resulting in lower bacterial migration to the knee joints, or to bacterial neutralization *in situ*. Therefore, 4C-Staph-immunized and mock-immunized mice were infected with the bioluminescent Xen36 strain and bioluminescence in the knee joints was monitored for 7 consecutive days, when mice were eventually sacrificed and CFUs in the knee joints quantified. Significant reductions of both CFU counts and bioluminescence were observed only at day 7 p.i in animals immunized with 4C-Staph (Fig. 6C and D), and not in the previous days (Fig. 6D, only day-1 is shown), clearly suggesting that the initial bacterial load in the joints was similar in mock-immunized and 4C-immunized mice, and the observed CFU reduction was the result of an efficacious local immune response.

### Immunization with 4C-Staph induced antigen-specific antibodies and recruitment of CD4+ effector memory T cells into the joints

**Figure 7:**
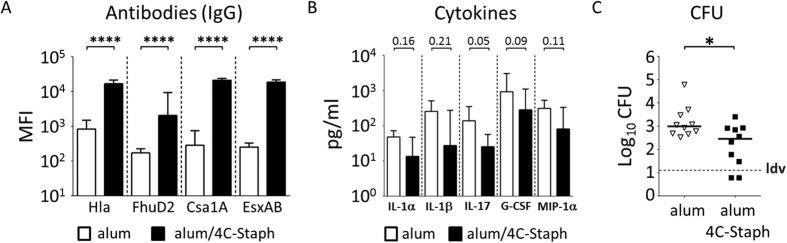
Antibodies induced by vaccination with alum/4C-Staph formulation reduce CFU counts in the joints of infected animals. (**A,B**) Antibody titration (Mean Fluorescent Intensity) against single combo antigens (**A**) and cytokine measurement (**B**) in knee joint washes of immunized mice 7 days after infection. Samples were collected from 9 to 10 animals/group from 2 independent experiments. Median and upper interquartile ranges are reported. (**C**) Log_10_ CFU counts of knee joint washes collected from passively immunized mice 7 days p.i. Single dots represent single animals form 2 independent experiments, black lines report median values for each group and the grey dotted line shows the lower detectable value (ldv). For all the data reported above, the two-tailed Mann-Whitney U-test was used to assess significance.

Antigen-specific antibodies were proven to play a central role in controlling *S. aureus* infection in different models^21^,^22^. We therefore assessed IgG titers specific for each single component of the 4C-Staph formulation in knee joint washes of mock-immunized and 4C-Staph-immunized mice sacrificed 7 days after the infection with Newman. As shown in Fig. 7A, significantly higher IgG levels against all components of the vaccine formulations were detected in lavages of alum/4C-Staph immunized mice, as opposed to washes from mice immunized only with the adjuvant, demonstrating that antigen-specific antibodies were induced by the immunization and were available in the joints. Alongside with this, a 3- to 10-fold drop in concentration of proinflammatory cytokines, such as IL-1α, IL-1β, IL-17, G-CSF and MIP-1α, was also measured in knee joint washes of animals actively immunized with 4C-Staph (Fig. 7B). This scenario might be compatible with the role played by specific functional antibodies in reducing the bacterial load and consequently the inflammatory state. To support this hypothesis, sera from rabbits immunized either with alum/4C-Staph or alum alone were passively transferred to mice, which were then infected with Newman and sacrificed 7 days later to measure CFU counts in joint washes. A statistically significant reduction of bacterial load was observed in mice passively immunized with 4C-Staph as compared with the negative controls (Fig. 7C).

**Figure 8:**
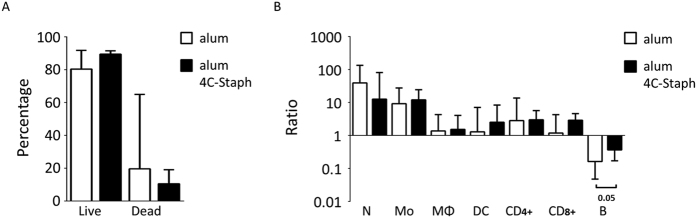
Immunization with alum/4C-Staph formulation results in partial restoring of B cell numbers in the knee joints of immunized mice. (**A,B**) Percentage of live and dead staining (**A**) and cellular recruitment analysis (**B**) in knee joint washes of immunized mice 7 days after infection. In (**B**), fold change of single cell sub-populations as compared to naïve mice is reported. Legend: N = neutrophils, Mo = monocytes; MΦ = macrophages; DC = dendritic cells; CD4+ = CD4+ T lymphocytes; CD8+ = CD8+ T lymphocytes; B = B lymphocytes. Medians and upper interquartile ranges are depicted from 3 independent experiments and 13–15 mice/group. The two-tailed Mann-Whitney U-test was used to assess significance.

**Figure 9:**
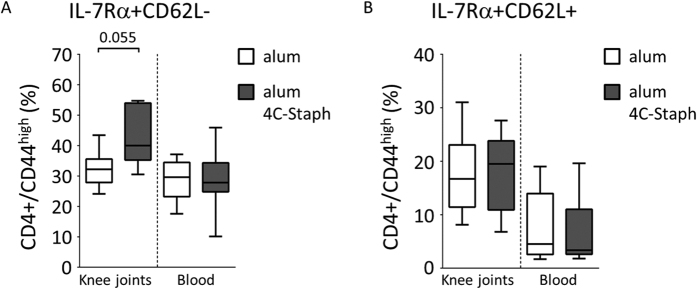
Knee joint recruitment of specific CD4+ Tem cells follows immunization with alum/4C-Staph combination. (**A,B**) IL-7Rα+CD62L- (CD4+Tem cells, **A**) and IL-7Rα+CD62L+ (CD4+ Tcm cells, **B**) percentage of CD4+/CD44^high^ T cells recruited into the knees and circulating in the blood of immunized mice after intravenous infection with *S. aureus* Newman strain. Five to 9 mice (knee joints) or 11 to 12 mice (blood) from 2/3 independent experiments were analyzed and median values together with interquartile ranges are shown in the box-whiskers graphs. The two-tailed Mann-Whitney U-test was used to assess significance.

To assess other potential protective mechanisms, we analyzed the immune cell populations recruited *in situ* in alum/4C-Staph-immunized mice and in the negative control group. No significant quantitative differences were observed neither between the percentages of live/dead cells (Fig. 8A), nor between the pattern of the major immune cell populations, with the exception of viable B cells for which a partial recovery was observed in immunized mice (Fig. 8B). However, when T lymphocytes from the two mouse groups were stained for CD3, CD4, CD44, IL-7Rα and CD62L to better characterize the T cell memory response^30^,^31^, an increase in the percentage of IL-7Rα+CD62L- cells (CD4+ effector memory, T_em_) was found in joint washes but not in the blood of mice immunized with 4C-Staph (Fig. 9A). No increase in the percentage of IL-7Rα+CD62L+ cells (CD4+ central memory, T_cm_) was found in joint washes or the blood of the same mice (Fig. 9B). Furthermore, when T_em_ cells were intracellularly stained for Th0, Th1 and/or Th2-indicator cytokines, significant increases of TNF-α and IL-4/IL-13 expression were observed in mice immunized with 4C-Staph as compared with the control (Table 1).

## Discussion

*Staphylococcus aureus* is the human bacterial pathogen most frequently associated with SA and OM, two severe infections of the joints and bones^5^,^7^,^16^,^20^. These infections and the related diseases are generally difficult to treat because they evolve very rapidly^4^ and often become chronic^13^. Ideally, antibiotic treatment of *S. aureus* infections should contribute to reducing arthritis and OM, but most antibiotics have been proven not to reach the joints and bones effectively^32^,^33^. Additionally, the number of multi-drug resistant *S. aureus* strains is presently increasing, which makes most antibiotics unusable^34^,^35^.

Given these premises, a vaccination strategy against *S. aureus* infection could have a positive impact on these diseases. This is also supported by the fact that the use of an effective vaccine against a major human pathogen responsible for SA, *H. influenzae type B*, resulted in the complete disappearance of this disease^6^. Although a vaccine against *S. aureus* is not yet available, we have recently demonstrated that immunization of mice with a multi-component protein-based vaccine (4C-Staph) was able to ameliorate the outcome of *S. aureus* infection in different *in vivo* models and that protection was dependent on both the humoral and cellular responses induced by vaccination^21^,^22^,^23^,^24^. We therefore set up a *S. aureus* infection model, resulting in a consistent bacterial infection of murine joints, in which we tested the ability of the proposed 4C-Staph formulation to reduce the burden of bacterial infection in the knees.

Mice have been considered good models for SA and OM^36^,^37^, and since blood is the most frequent source of infection in humans^7^,^37^, efforts were made to set up and characterize an acute hematogenously-derived murine model of arthritis^25^,^28^,^38^,^39^,^40^, with most of the reports being concentrated on observations made in the paws^36^,^37^. Recently, also thanks to the great improvement in the preclinical imaging, other groups focused their attention in the field and were able to set up long lasting models of OM which evolved from an acute to a chronic phase^26^,^41^,^42^.

We believe that the model described here provides additional and relevant information in the field for different reasons: (i) we concentrated on *S. aureus* infections of the knees which, together with the hips, are the joint sites most frequently infected in humans^7^,^43^. Preferential infection of the knees in mice injected intravenously was confirmed using bioluminescent *S. aureus* strains belonging to different capsular serotypes, and we showed that the pathogen infiltrates the murine joints very soon after infection and colonizes the site (Fig. 1A and Fig. S1); (ii) the observation of mice was extended as long as 3 months p.i., and we consider this may be relevant since *S. aureus* infection in humans results in a local and persistent inflammatory response, which can eventually cause or facilitate chronic disease in the joints^7^,^44^. Interestingly, this outcome was actually confirmed in infected mice by histopathological analysis of the knee joints (Fig. 2 and Supplementary Table S1), suggesting that the murine model somehow mimics the progression of the disease in the human host, as already stated for OM^26^; (iii) multiple parameters were quantitatively measured (CFUs, cytokines, immune cell recruitment, antigen-specific antibodies), determining the bacterial burden as well as host inflammatory and immune responses following infection and/or vaccination; (iv) both systemic and local inflammatory and immune responses were analyzed. By doing so, differences were appreciated and potential infection- and vaccination-specific signatures were identified.

The prolonged observation of infected mice allowed us to distinguish among different phases. During the first two weeks of infection, bacteria rapidly proliferated both in the joints and MIP-1 in kidneys (Fig. 3), which were our benchmark of systemic infection^45^, and about ten percent of the mice died (Fig. 3D). Evident signs of inflammation were observed in the joints and bones of all infected mice (Fig. 2A and B), and strong proinflammatory (IL-1, IL-6, TNF-α) and chemoattractant (G-CSF, GM-CSF, KC/IL-8, MCP, MIP-1α/β) responses were found both in the joints (Fig. 4A) and systemically (Fig. 5A). Changes occurred in the immune cell repertoire (Figs 4B and 5B), and pathogen-specific antibodies were measured both in the blood and the synovial fluid (Fig. 3E and F). Some of these findings had already been published elsewhere, even if using different mouse and/or bacterial strains^26^,^46^. This early immune response possibly correlates with the control of bacterial burden observed in the knees and kidneys (Fig. 3A and B), the highly reduced mortality observed two months p.i. (Fig. 3D), inflammation reduction and lower cellular recruitment in the knees (Fig. 4A and B), and a mild severity of disease (Fig. 2A and B).

However, monitoring infection progression over time revealed different scenarios. Most markers of inflammation at the joints, but also systemically, were almost or completely back to basal levels 1–3 months p.i. (Fig. 4A), as partially observed by L. Tuchscherr and collegues, both *in vitro* and *in vivo*^41^. Nevertheless, arthritis, and OM in particular, worsened dramatically (Fig. 2A and B). Local persistency of an inflammatory condition might have contributed to transforming the joint area into an isolated niche hardly reachable by antibodies, which in turn could have a negative impact on the physiological and positive action of the host immune system (Fig. 3F). This is actually observed in humans where joint drainage is often needed in order to reduce the internal pressure that blocks cellular recruitment^7^.

At the latest time point, a systemic analysis of bacterial burden showed indeed that not only bacteria were still present in joints and kidneys, but also that they could be measured for the first time in blood (Fig. 3C), possibly indicating abscess disruption and systemic diffusion of the infection^47^. Consistently with this observation, a sharp decrease of mice survival was observed from 60 days p.i. onwards (Fig. 3D), in spite of the high level of serum IgG specific for *S. aureus* antigens (Fig. 3F), and elevated neutrophil levels in the blood (Fig. 5B). These observations strongly suggest that *S. aureus*-induced immune priming is not able to contain the infection.

The analysis of the inflammatory reaction to the infection revealed differences among the markers measured systemically in the blood and locally at the joints. A set of cytokines were present only in the serum (IL-5, IL-10, IL-12p70, MIP-1β, TNF-α Fig. 5A), while others only in the joints (IL-9, MIP-1α, Rantes, Fig. 4A). Similarly, the Th proinflammatory response was Th1/Th17-like in the joints (IL-1, IL-6, IL-12, INF-γ, IL-17), and more complex in the serum with the presence also of Th2-like (IL-5, IL-13) and Treg-like (IL-10) cytokines. The observation that IL-10 was measured in the blood throughout all the 3 months of observation but not in the joints of infected mice may be related to disease development, since suppression of IL-10 activity could at least partly account for the intense and persistent inflammatory state that is a pre-condition for the development of arthritis^48^,^49^,^50^,^51^,^52^. This may be consistent with the knowledge that *S. aureus* and other pathogens secrete enzymes that can degrade specific cytokines^53^,^54^. Bacterial-dependent degradation of host factors may also explain why the only three cytokines detectable in the joints of naïve mice (IL-9, IL-13 and eotaxin), and possibly involved either in cartilage homeostasis^55^ or in processes of the host immune response^56^,^57^, decreased very early during infection, which could somehow favour *S. aureus* colonization of the infected joint. Noteworthy, it was previously reported in a similar model that IL-10 in the sera of infected mice peaked 2 weeks after infection and then decreased to basal level^41^. This difference may be due to the different strain of mouse and/or the different strain of *S. aureus*^58^ used.

Almost all immune cell types were recruited in the knee joints very early after infection (Fig. 4B). Neutrophils were by far the most abundant cell population, as we had previously observed in other local infection models^22^. Whether recruited neutrophils and monocytes were in a pro-inflammatory or anti-inflammatory activation state has not been assessed in this study. It has been reported that chronic activation could predisposes the cells to convert into myeloid-derived suppressor cells (MDSC) with an anti-inflammatory phenotype, which has been repeatedly shown to worsen the outcome of staphylococcal infection^59^,^60^. Interestingly, B cell numbers decreased rapidly, remaining under basal level for at least 2 weeks. A similar reduction, as compared with CD4+ and CD8+ T lymphocytes, was also observed for B cells in the blood (Fig. 5B). It was previously reported that B cells do not play a major role against *S. aureus* infections^38^,^61^. On the contrary, our results seem to directly correlate decrease of B cell number in the joints and blood with the *S. aureus* early infection phase, suggesting that the pathogen might be directly involved in suppressing B cell responses systemically and in the joints, through different known mechanisms^39^,^62^,^63^, and that this suppression might cause underestimation of the effective role of these cells in joint infections, as recently postulated in other models^64^. Nevertheless, since those studies have been conducted in BALB/c inbred mice using a different bacterial strain, we cannot exclude that the different phenotypic background of mice and/or bacteria may have influenced, at least partially, this outcome, as it has been well described in a systemic infection model^58^.

Once the infection model was characterized, the following question was whether and to what extent could a *S. aureus* vaccine formulation positively affect the outcome of the infection. We did not attempt to quantify the protective effect of the vaccine by histopathological examination. In fact, it would have been difficult to carry out a quantitative analysis because the samples to be analyzed included different tissues (i.e. skin, muscle, tendons, cartilages and bones), which respond differently to fixation and slice preparation, and require demineralization in order to allow bone slicing. For this reason the analyses that we performed were typically qualitative and limited to confirming the pathological state of the infected mice without taking into account the possible effects of the vaccine.

We have previously shown that the 4C-Staph formulation is effective in reducing *S. aureus* systemic infection in different murine models^21^,^22^,^23^,^24^. Immunization of mice with this formulation was associated in the present model with reduction of the bacterial load both in knee joints and kidneys (Fig. 6A and C), mitigation of the general inflammatory condition (Fig. 7B) and, in particular, decreasing levels of IL-17, which have been shown to be directly involved in the development of arthritis after bacterial infection^65^. Interestingly, the protection experiment performed with the bioluminescent Xen36 strain suggested that local reduction of bacteria observed in the joints was dependent on the induction of an efficacious local immune response, rather than to the lower number of bacteria reaching the joints due to systemic neutralization (Fig. 6C and D). The induction of both humoral and cellular immune responses in the joints might account for the reduction of bacterial burden. Antibodies do play a central role in defeating *S. aureus* mediated diseases^66^,^67^, and the 4C-Staph formulation was shown to induce a potent antibody response against all components^21^. Here we show that antibodies specific for *S.aureus* can reach the joints where they are likely to play a role in reducing the local bacterial burden, as suggested by passive transfer experiments (Fig. 7C).

Remarkably, immunization with 4C-Staph had a positive effect on the composition of the immune cell repertoire as well. Two major changes were observed. The number of B lymphocytes remained significantly higher in the joints of 4C-Staph-vaccinated animals compared to alum alone vaccinated ones (Fig. 8B), possibly due to the inhibition of α-toxin mediated cytotoxicity^68^,^69^. This may positively affect the control of the infection not only because a greater number of viable B cells is present *in situ* and produces specific IgG, but also because the antigen presenting activity may be exerted more efficiently^70^. The second change that we observed concerned the specific CD4+ T cells induced by vaccination with 4C-Staph, and which we have previously shown to protect mice against septic death^23^,^24^. In fact, we observed that, in mice immunized with the 4C-staph formulation, a significantly higher number of CD4+ T effector memory lymphocytes was present in the knee joints as compared to the blood, and that these cells were mainly associated with the presence of Th0-Th2 but also of Th1-like cytokines, as was to be expected on the basis of the alum adjuvant used (Fig. 9A and Table 1). This finding is intriguing considering that memory Th1 cells have been shown to be protective in an invasive mouse model of *S. aureus* infection^71^ and that patients with CD4+ T lymphocyte disorders (i.e. HIV patients) undergo recurrent infections more frequently^72^,^73^, suggesting that these cells might play a role in controlling *S. aureus* mediated infections also in humans. In this context, the finding that immunization induces CD4+ effector memory T cells, which migrate into peripheral tissues and organs where they might contribute to mounting an effective immune response against *S. aureus* would represent an added value for the proposed 4C-Staph vaccine formulation^74^,^75^,^76^.

In the present study we did not address the possible role of *S. aureus* biofilm in infection models of SA and OM and how it could impact the effectiveness of the vaccine formulation that we tested. All the studies which investigated this issue used infection models based either on bone implants which were previously coated with bacteria^77^,^78^,^79^ or with direct deposition of bacteria on bones^80^,^81^. In both cases biofilm formation *in situ* and, therefore, its analysis and quantification was facilitated by the experimental approach used. On the other hand, as we know, nothing has been reported instead on biofilm formation in hematogenous infection models of SA and OM and this is probably due to the objective difficultness to deal with this issue in a more complicated model of systemic infection. Moreover, in this type of model the choice of the strain used for the infection may be more critical, and using the poor biofilm former Newman strain^82^ rather than other strains may determine different outcomes, making it difficult to predict if, how and to what extent could biofilm formation affect this specific model. Of course we cannot rule out the possibility that biofilm formation may have occurred in the model that we described here, which could have affected the measurement of some of the parameters that we described (expecially at longer time points), but for the reasons mentioned above, we believe that this should be addressed in a separate study, possibly using different infective *S. aurues* strains and different technologies.

On the other hand, since we mainly focused on the response to the vaccine formulation and on the identification of specific immune traits in the first week after the infection, in other words during the acute phase of the disease, we expect initial biofilm formation to play a minor role in negatively affecting vaccine effectiveness in hematogenous-derived disease.

In conclusion, we believe that the model we have set up and characterized enriches the present knowledge on animal models of joint infection that attempts mimicking the human disease condition. A model of hematogenously derived *S. aureus* knee joint infection is now available which, similarly to humans, causes a severe and persistent host inflammatory response that sets the stage for progression towards chronic arthritis. Secondly and importantly, this model highlights that systemic and local inflammatory and immune responses elicited by host infection and/or vaccination can be distinguished. The knowledge that specific immune traits can be followed may be particularly useful when evaluating a vaccine formulation against *S. aureus*, a pathogen that exerts its pathogenic potential in the joints as well as in a number of diverse biological niches.

## Methods

### Bacterial strains and preparation of bacteria

*S. aureus* Newman strain and bioluminescent *S. aureus* Xen36, Xen8.1, Xen29 (Perkin Elmer) were used here. *S. aureus* strains were grown in tryptic soy broth (TSB) from an initial Absorbance at 600 nm (A_600_) of 0.05 in a standard 1-cm cuvette. Bacteria were grown as previously reported^22^.

### Mice immunization and infection

All animal studies were carried out in compliance with current Italian legislation on the care and use of animals in experimentation (Legislative Decree 26/2014) and with the Animal Welfare Policy and Standards. Protocols were approved by the Italian Ministry of Health (authorization 185/2011-B) and by the local Animal Welfare Body (AWB authorization 201105). After infection, mice were monitored daily and euthanized when they exhibited defined humane endpoints pre-established in agreement with internal Animal Welfare Policies.

Ten-weeks-old CD1 female naïve mice were used in these studies. When immunization was required, 5-weeks-old CD1 female mice were used. Animals were actively and passively immunized as previously described^21^,^22^. Thirteen days after the last immunization, mice were injected in the lateral tail vein as described elsewhere^21^ using an infective dose of about 2E + 06 CFU/mouse for the Newman strain. The same dose was also used to infect naïve animals. The infective dose for the Bioluminescent strains was about 1–2E + 07 CFU in 100 μl of PBS instead. Animals were finally sacrificed at pre-established time points to collect the blood, kidneys and hind legs. Kidneys were homogenized using the gentleMACS™ Dissociator, Miltenyi Biotech, following supplier’s instructions. To perform knee joint washes, the skin was removed and the hind legs were dissected. Muscles all around the knee area were excised in order to expose the knee and an incision was precisely made with a disposable scalpel between the femur and tibia to expose the joint. To avoid inclusion of surrounding tissues, the knee was exposed the minimum necessary to proceed with the wash. When abscesses from surrounding tissues were observed, either they were not included in the wash or, if this was not possible, the sample was not collected. Once the knee was exposed, it was first embedded several times into a well of a 6-well plate containing 3 ml of RPMI 1640 medium, then 5 washes using 1 ml of the same medium were done directly in the knee. The resulting suspension was filtered with a 70 μm nylon mash (Becton Dickinson) and data obtained were presented as analyte/wash. The remaining wash was centrifuged to separate cells from supernatant, which was filtered through a 0.22-μm-pore-size filter and stored at −20 °C for antibody titration and cytokine profiling. Cytokine analysis was performed within a few weeks post collection to avoid degradation. Knee joint washes and heparinized blood samples were also analyzed to identify and measure the different immune cell populations. Filtered joint washes were directly analized by Flow Cytometry. Blood samples were first treated with the BD Pharm Lyse™ solution to lyse red blood cells, centrifuged at 500 × g for 5 min, washed in PBS and finally resuspended in an opportune volume for Flow Cytometry staining.

### *In vivo* imaging

For the 2D and 3D *in vivo* imaging acquisitions, IVIS^®^ 100 and IVIS^®^ SpectrumCT (Perkin Elmer) were used, respectively. Isofluorane at a concentration of 2.5% was used to anhestetize animals. Bioluminescent images were displayed using a pseudocolor scale (blue representing the least-intense light and red representing the most-intense light) that was overlaid on a gray-scale image to generate a 2D or 3D picture of the localization of bioluminescent bacteria in the animal. Photon emissions from a region of interest (ROI) were quantified using the Living Image 3.2 software package (Perkin Elmer) following manufacturer’s procedures, and the data were presented as relative light units contained within each region (photon/second/cm^2^/sr).

### Histopathological and immunohistochemical examination

Histologic examination of the hind legs was performed after standard 4% buffered formaldehyde fixation, decalcification, paraffin embedding, and staining with hematoxylin and eosin. A blind assessment of severity and stage of SA/OM was performed by an experienced pathologist who analyzed the different features of the joint and bone (exudate, synovitis, pannus formation, cartilage, bone destruction and deformation, abscess formation, presence of different characteristic cells). Each slide was graded using a specific scale. Score legends: Severity = 0) normal, 1) mild, 2) moderate, 3) severe, 4) very severe, 5) extremely severe; stage = 0) normal, 1) iper-acute, 2) acute, 3) sub-acute, 4) chronic. Immunohistochemical staining on 4-μm knee joint sections was performed by using Ventana Discovery^®^ XT autostainer. After deparaffinization, heat-induced antigen retrieval was performed in acidic buffer (Cell Conditioning Solution 2, Roche 950-123). Slides were then treated with high ionic strength protein blocking solution for reduction of background (Antibody Block Roche 760-4204). Primary anti-*S. aureus* (Abcam ab69534) or anti-neutrophil (Abcam ab56313) antibodies conjugated with biotin were diluted to 4.5 μg/ml in Antibody Diluent (Roche 760-108) and allowed to react with the slides overnight at room temperature. Negative control slides with isotype control antibody were included. The DISCOVERY^®^ RedMap Kit (Roche 760-123) and BlueMap Kit (Roche 760-120) were used for detection of anti-*S. aureus* and anti-neutrophils, respectively.

### Immunofluorescence and confocal microscopy

Knees from 10-week-old mice infected with *S. aureus* Newman strain were frozen and cut using a cryostat. Samples were fixed in 2% formaldehyde for 20 min at room temperature (RT) and then washed three times with a permeabilizing solution containing Bovine Serum Albumin (3%) and saponin (1%). Samples were treated with an antibody mix containing chicken anti-goat conjugated with ALEXA FLUOR 647 (far red, final dilution 1:200 to stain osteocalcin), goat anti-rabbit conjugated with ALEXA FLUOR 488 FITC (green, final dilution 1:200 to stain *S. aureus* cells), and phalloidin (red, final dilution 1:300, conjugated with ALEXA FLUOR 568 to stain the muscle actin) for 1 h, washed three times with the permeabilizing solution (BSA 3% and saponin 1%), twice with PBS and twice with water. Slides were finally mounted using mounting medium with DAPI (Life Technologies) and incubated overnight at RT, and images were obtained using a Zeiss LSM 700 confocal microscope (Carl Zeiss).

### Serological analysis, Cytokine profiling and immune cell staining in knee joint washes and blood

Cell suspensions from knee joint washes and heparinized blood were prepared as reported above and stained as previously described^22^. Serological analysis and cytokine profiling were performed as previously described^22^. Antibodies used for the staining are the following: Ly6C-FITC, Ly6G-PE, CD11b-APC, CD4-V500, CD3-PE-Cy7 (BD Biosciences), F4/80-V450, CD11c-APC-eFluor780, CD19-PE-Cy5 (eBioscience), and CD8-PE-TR. For the intracellular staining of CD4+ T cells, cell suspensions were prepared as above then washed twice with PBS and resuspended in Live and Dead Near-IR (Invitrogen) 1/1000 for 20 min in the dark at RT. Cells were then washed again in PBS and resuspended in PBS-BSA (Sigma) 1% + CD16/CD32 (FcγIII/II Receptor, BD Pharmingen) to saturate aspecific binding sites, and incubated for 20 min in the dark at RT as specified by manufacturer’s instructions. 1/1 volume of antibody mix containing CD62L-A700 (BD biosciences) and CD127-PE (eBiosciences) in PBS-BSA 1% was then added and incubation was prolonged for an additional 20 min at RT in the dark. Samples were then washed twice with PBS and resuspended in CITOFIX/CITOPERM^TM^ (BD) following manufacturer’s procedure. Cell suspensions were then washed in Perm/Wash^TM^ buffer (BD) following manufacturer’s procedures and resuspended in the same buffer with a mix of antibodies (CD3-BV605, CD4-V500, CD44-V450, TNF-α-Alexa488 (BD Biosciences), INF-γ-BV785, IL-2-PE-Cy5 (BioLegend), IL-4/IL-13-PerCP-eFluor710, IL17-PE-Cy7). Incubation was done for 20 min at RT in the dark, then cells were washed once in Perm/Wash^TM^ and once in PBS and finally resuspended in PBS + BSA1% for FACS analysis. Acquisition of labeled cells was performed using a FACS LSRII special order cytometer (Becton Dickinson). BD TruCount^TM^ Absolute counting tubes were used to determine the absolute cell number for each cellular population, according to manufacturer’s instructions. Only samples with at least 25 CD4+/CD44high/IL-7Rα+CD62L- were considered. Gating strategies are reported in supplementary material, Figures S2 and S3.

### Statistics

Statistical analyses were performed using Graph Pad Prism 6. The Mann-Whitney U-test (two tailed), the Fisher’s exact test (two tailed) or the Spearman correlation analysis were used to calculate statistical significance. P values of <0.05 were considered statistically significant. Legend: *p-val < 0.05; **p-val < 0.01; ***p-val < 0.001; ****p-val < 0.0001.

## Additional Information

**How to cite this article**: Corrado, A. *et al*. *Staphylococcus aureus*-dependent septic arthritis in murine knee joints: local immune response and beneficial effects of vaccination. *Sci. Rep.* **6**, 38043; doi: 10.1038/srep38043 (2016).

**Publisher's note:** Springer Nature remains neutral with regard to jurisdictional claims in published maps and institutional affiliations.

## Supplementary Material

Supplementary Information

## Figures and Tables

**Table 1:** Effector memory CD4+ T cells recruited into the knee joints of vaccinated mice express mainly Th0-Th2-like cytokines.

	Th0-like cytokines		Th1-like cytokines	Th2-like cytokines	Th17-like cytokines
Sample	IL-2+ (%)	TNF-α+ (%)	IFN-γ+ (%)	IL-4/IL-13+ (%)	IL-17+ (%)
alum^a^	2.89	10.94	6.41	11.70	3.68
alum/4C-Staph^b^	5.17	17.47	9.62	18.71	3.94
Fisher’s exact test	0.067	**	0.072	**	ns

^a^Pool of 9 animals from 2 independent experiments.

^b^Pool of 5 animals from 2 independent experiments.
