# Supplementary material for: Staphylococcus aureus-dependent septic arthritis in murine knee joints: local immune response and beneficial effects of vaccination
Source: Sci Rep. 2016 Nov 30;6:38043. doi: 10.1038/srep38043 (PMC5128924; doi:10.1038/srep38043)
Supplement: Supplementary Information [file srep38043-s1.pdf]

***Staphylococcus aureus*-dependent septic arthritis in murine knee joints: local immune response and beneficial effects of vaccination**

Alessia Corrado[1,2], Paolo Donato[1], Silvia Maccari[1], Raffaella Cecchi[1], Tiziana Spadafina[1,3], Letizia Arcidiacono[1], Simona Tavarini[1], Chiara Sammicheli[1], Donatello Laera[1], Andrea Guido Oreste Manetti[1], Paolo Ruggiero[1], Bruno Galletti[1], Sandra Nuti[1], Ennio De Gregorio[1], Sylvie Bertholet[1], Anja Seubert[1], Fabio Bagnoli[1], Giuliano Bensi[1] and Emiliano Chiarot[1]

Author affiliation

[1] GSK Vaccines, Via Fiorentina 1, Siena, Italy 53100

[2] Present address: Divisions of Pulmonary, Allergy, & Critical Care Medicine, Emory University, Atlanta, GA 30322

[3] Present address: Fondazione Achille Sclavo ONLUS, Via Fiorentina, 1, Siena, Italy 53100

Corresponding author

Giuliano Bensi, GSK Vaccines Srl, Via Fiorentina 1, Siena, Italy 53100. Telephone: +39 0577 245231; Fax: +39 0577 243564

Email: giuliano.x.bensi@gsk.com

Condensed Title

S. aureus-dependent septic arthritis in mouse knees

Supplementary information

Supplementary figure S1

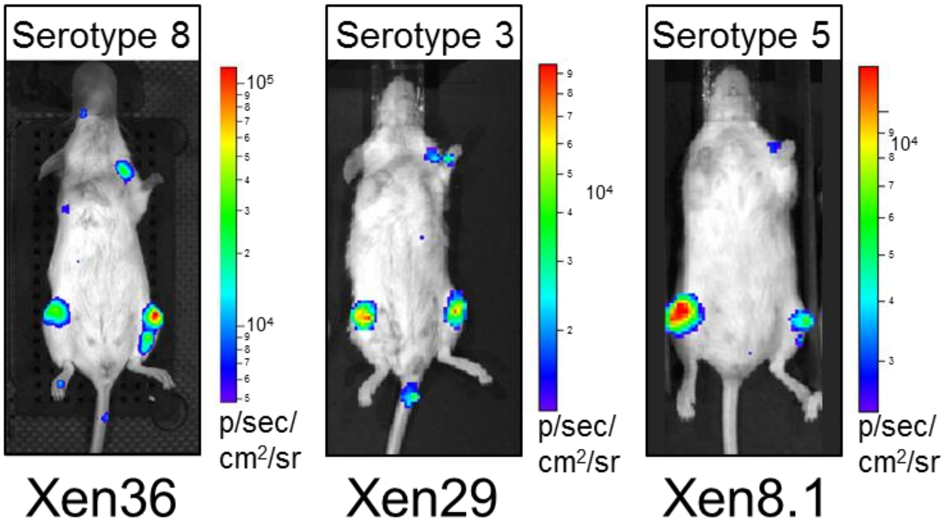

Supplementary figure S1. *S. aureus* tropism for bones and joints is not dependent on capsular polysaccharide. Ventral 2D acquisitions of CD1 mice infected with bioluminescent *S. aureus* strains belonging to different capsular serotypes. Mice intravenously infected with *S. aureus* Xen36 (serotype 8), Xen29 (serotype 3) or Xen8.1 (serotype 5) were photographed 2-7 days after infection and 1 animal out of 5 is reported for each strain.

Supplementary figure S2

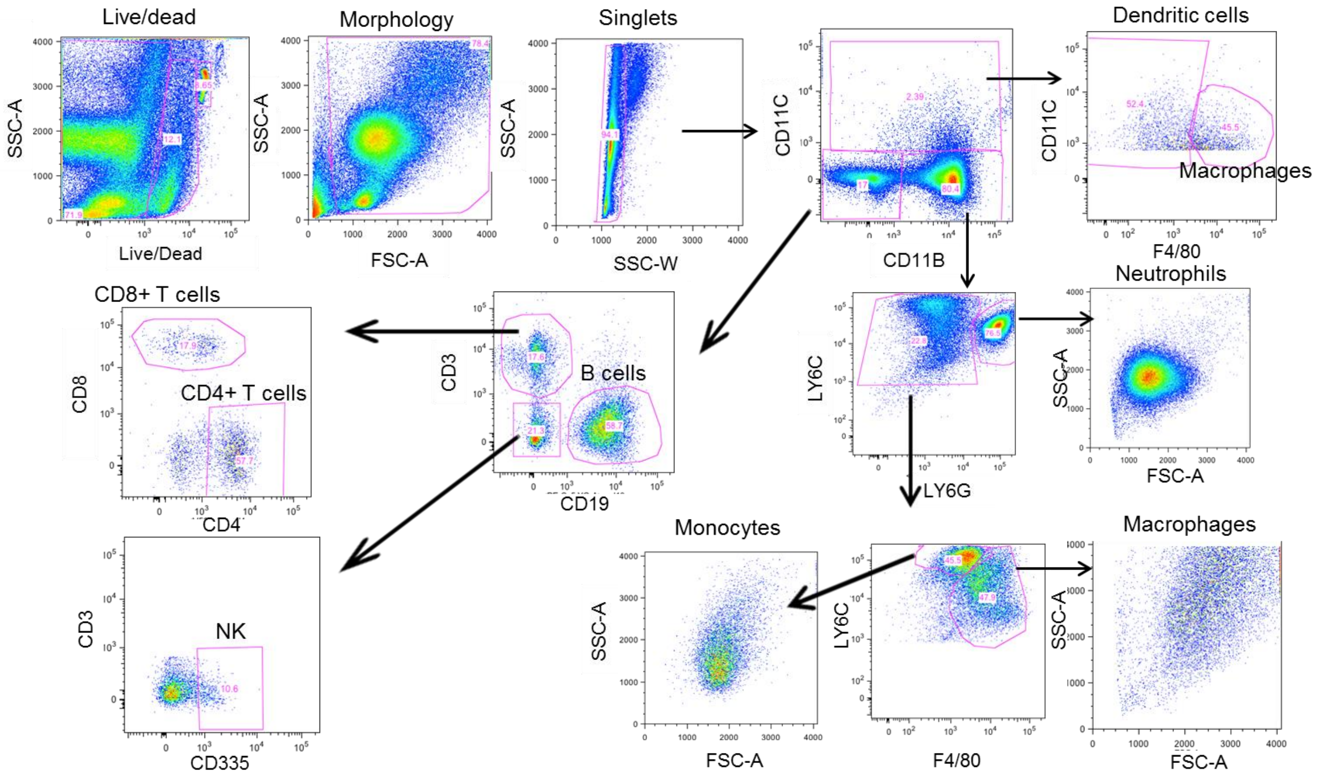

Supplementary figure S2. Gating strategy for the analysis of immune cells in mouse knee joint washes and blood. The flow cytometry analysis was used to identify different immune cell subsets both in knee joint washes and the blood of naïve and *S. aureus* intravenously infected animals. On the X and Y axes are reported the different markers utilized for the specific analysis. Pink gates identify the different cell subsets analyzed.

Supplementary figure S3

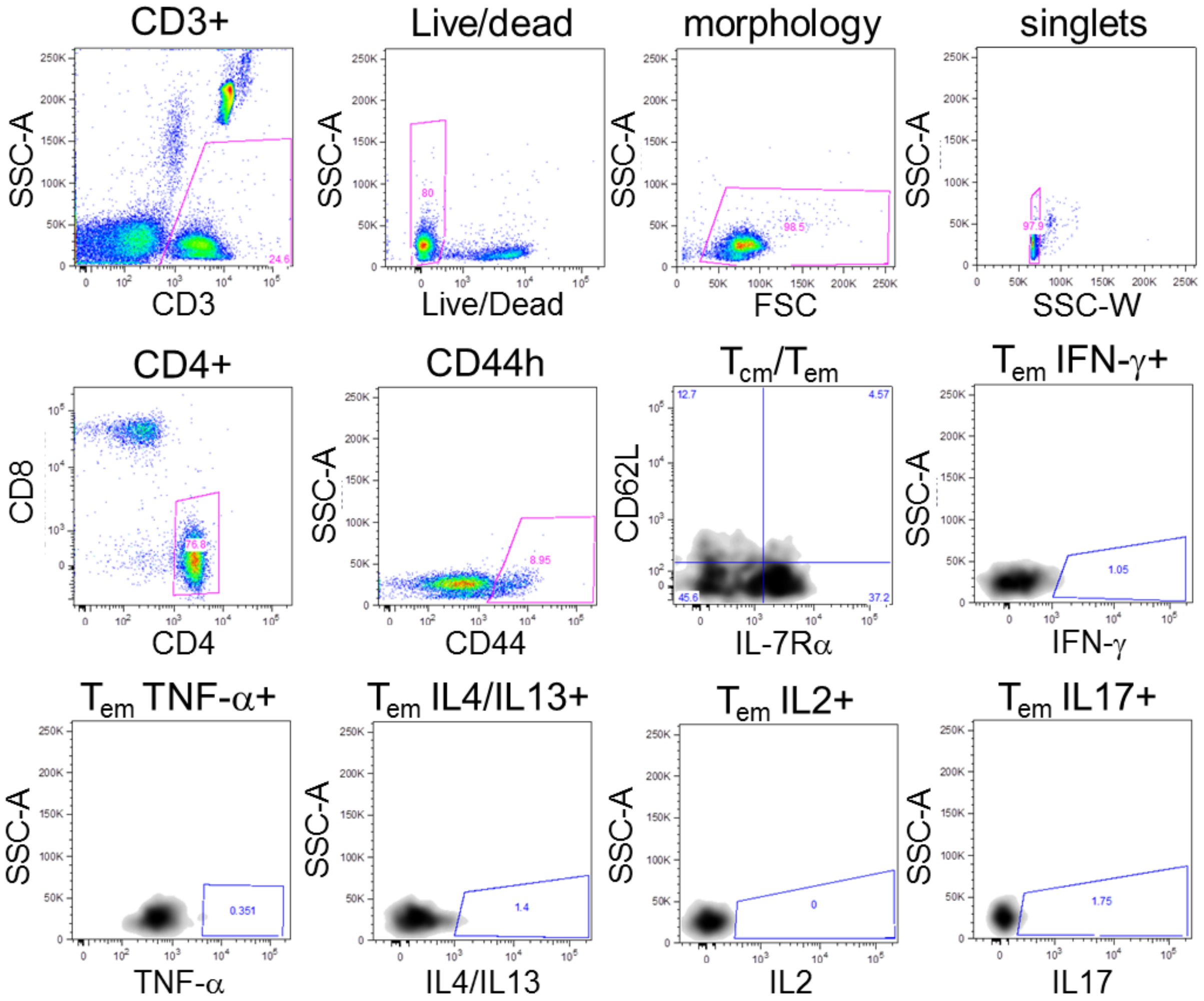

Supplementary figure S3. Gating strategy for the analysis of CD4+/CD44high/IL-7Rα+CD62L- and CD4+/CD44high/ IL-7Rα +CD62L+ T cells. The flow cytometry analysis was used to stain different CD4+ T cell subsets and the strategy is reported above with a typical example of the staining of a sample (knee joint lavage). The same analysis was applied to both knee joint washes and the blood of infected animals.

Supplementary table S1: Histopathological examination of the knee joints from single mice

Days after infection	Severity Grade	Osteomyelitis	Osteomyelitis femur	Osteomyelitis tibia	Arthrosynovitis	Myositis	Bone destruction	Abscess	Fibrosis (moderate)	Fibrosis (severe)
3	0	-[a]	-	-	-	-	-	-	-	-
3	0	-	-	-	-	-	-	-	-	-
3	0	-	-	-	-	-	-	-	-	-
3	0	-	-	-	-	-	-	-	-	-
3	0	-	-	-	-	-	-	-	-	-
3	0	-	-	-	-	-	-	-	-	-
3	0	-	-	-	-	-	-	-	-	-
3	1	X[b]	X	-	-	-	-	-	-	-
7	0	-	-	-	-	-	-	-	-	-
7	0	-	-	-	-	-	-	-	-	-
7	2	-	-	-	-	X	-	-	-	-
7	0	-	-	-	-	-	-	-	-	-
7	2	X	X	-	X	-	-	-	-	-
7	2	X	X	-	X	X	-	X	-	X
7	4	X	X	-	X	X	X	X	-	X
7	3	X	X	X	X	X	X	X	X	
14	0	-	-	-	-	-	-	-	-	-

**14**	**0**	-	-	-	-	-	-	-	-	-
**14**	**3**	X	X	-	-	-	-	X	X	-
**14**	**2**	X	X	-	-	X	X	X	X	
**14**	**4**	X	X	-	X	X	X	X	-	X
**14**	**3**	X	X	-	X	X	X	X	-	X
**14**	**3**	X	X	X	X	X	X	X	-	X
**30**	**0**	-	-	-	-	-	-	-	-	-
**30**	**0**	-	-	-	-	-	-	-	-	-
**30**	**0**	-	-	-	-	-	-	-	-	-
**30**	**0**	-	-	-	-	-	-	-	-	-
**30**	**2**	X	X	-	-	X	X	X	X	-
**30**	**3**	X	X	X	-	X	X	X	X	-
**30**	**3**	X	-	X	X	X	X	X	X	-
**30**	**5**	X	X	-	X	X	X	X	-	X
**90**	**0**	-	-	-	-	-	-	-	-	-
**90**	**4**	X	X	-	-	X	X	X	-	X
**90**	**5**	X	X	X	-	X	X	X	-	X
**90**	**5**	X	X	X	-	X	X	X	-	X
**90**	**5**	X	-	X	X	X	X	X	-	X
**90**	**4**	X	X	-	X	X	X	X	X	-

[a] = negative

[b] = positive

Veterinary report (addendum to supplementary table S1)

FFPE, decalcified samples of one of the hind legs (right or left) including the knee, femur, tibia and fibula (the latter not always present in the section due to orientation artefacts) and regional soft tissues were examined for each time point.

Inflammatory changes were present overall in 19 out of 37 samples (1 out of 8 after 3 days, 4 out of 8 after 7 days, 5 out of 7 after 14 days, 4 out of 8 after 30 days and 5 out of 6 after 90 days p.i.). In all cases the inflammation was pyogranulomatous to mixed, and ranged from mild to severe. Macrophages, PMNs/neutrophils, lymphocytes and occasional plasma cells were seen in mixed inflammatory exudates, while in pyogranulomatous exudates, macrophages and PMNs predominated. Macrophages were always abundant, with marked signs of cellular activation (loose chromatin patterns, prominent nucleoli and foamy cytoplasm). Severity of lesions was arbitrarily classified within the following ranges: 0 = no inflammation to 5= most severe inflammation, depending on extension of lesions. Grade 1 severity was seen in 1 case (3 days p.i.), while grades 4 and 5 were seen at 30 days p.i. (1 case out of 4) and 90 days p.i. (5 cases out of 5).

Pyogranulomatous or mixed, mild to severe arthrosynovitis was seen in the totality of cases presenting osteomyelitis for 7-day-old samples, while the percentage of affected legs was 60%, 50% and 40% in 14-day-, 30-day- and 90-day old samples, respectively.

Abscesses were seen in 17 out of 19 cases of osteomyelitis and always associated to myositis and bone destruction in lesions aged 30 days and over. Abscesses often contained a central area of eosinophilic, homogeneous material stippled with cellular debris (necrosis) and hyper-eosinophilic or sometimes basophilic amorphous, irregularly shaped core (bacterial nidus), the latter often cuffed by necrotic PMNs.

Bone destruction features included focal bone necrosis, localized to extensive lamellar destruction to complete obliteration of tissue.

In most samples (17 out of 19, 100% of cases aged 14 days or above), elongated cells were seen, often surrounded by homogeneous eosinophilic material consistent with fibroblasts/fibrocytes and collagen. These cells were sometimes admixed to the inflammatory exudate, while they often formed bundles that encircled foci of inflammation or abscesses.
